# Exploring the Perspectives of Patients and Healthcare Providers on Rheumatology Clinical Trials: A Single-Center Cross-Sectional Study in Hungary

**DOI:** 10.3390/jcm15072547

**Published:** 2026-03-26

**Authors:** Monika Bodoki, Erzsébet Hunyadi, Andrea Domján, Katalin Hodosi, Zoltán Szekanecz, Nóra Bodnár

**Affiliations:** Department of Rheumatology, Faculty of Medicine, University of Debrecen, 4032 Debrecen, Hungary; bbereczki.erzsebet@gmail.com (E.H.); domjan.andrea@gmail.com (A.D.); khodosi@gmail.com (K.H.); szekanecz@gmail.com (Z.S.); drbodnarnora@gmail.com (N.B.)

**Keywords:** clinical trial, patient satisfaction, patient motivation, quality of healthcare

## Abstract

**Objectives:** Clinical trials are essential for therapeutic innovation in rheumatology. A recent decline in clinical trial activity in Hungary has highlighted the need to better understand patient experiences and motivations. This study assessed patient satisfaction and motivation in clinical trials, compared these with routine specialist care, and evaluated healthcare professionals’ motivations. **Methods:** In this single-center, cross-sectional study, 129 patients completed self-administered questionnaires (61 trial participants and 68 receiving routine care) primarily using a 6-point Likert scale; additionally, 21 healthcare professionals rated their motivations on a Visual Analog Scale (VAS 0–10). Categorical variables were analyzed using chi-square or Fisher’s exact tests, and continuous variables using paired two-tailed *t*-tests. **Results:** The main drivers of trial participation were physician recommendations (100%) and trust in the treating physician (100%). Access to novel therapies (98%), closer monitoring (83%), and additional diagnostic procedures (95%) were also significant motivators. Trial participants reported significantly higher satisfaction compared with routine care in terms of consultation time (97% vs. 36%, *p* < 0.001), staff availability (95% vs. 41, *p* < 0.001), assistance (93% vs. 36%, *p* < 0.001), and visit organization (98% vs. 34%; *p* < 0.001). Overall satisfaction with routine care remained high in both groups. In the control group, fears of disease worsening and the burden of frequent visits were key deterrents. Among healthcare professionals, access to innovative treatments was the strongest motivator, while administrative workload and documentation demands were the primary barriers. **Conclusions:** Clinical trial participation is associated with high patient satisfaction, driven by physician–patient trust and structured, personalized care. Reducing administrative burdens may be crucial for sustaining clinical research in academic settings.

## 1. Introduction

In the context of this study, clinical trials are defined as prospective research investigations involving human participants, designed to evaluate the safety, efficacy, and pharmacological properties of novel therapeutic interventions [1]. These trials are conducted in accordance with the International Council for Harmonisation (ICH) Good Clinical Practice (GCP) guidelines and the Declaration of Helsinki. Clinical trials are fundamental to therapeutic innovation, particularly in rheumatology, where rapid advances in targeted and biologic therapies have substantially transformed patient outcomes. The Department of Rheumatology and Immunology, Clinical Centre, University of Debrecen, has actively participated in clinical research for several decades, with a sustained commitment to patient-centered care and high-quality trial conduct. Since 2001, our site has been a cornerstone of this effort, becoming one of the first accredited investigational sites in Hungary. Our team has played a pivotal role in the clinical development of nearly all biological and targeted therapies currently utilized in rheumatology. Currently, the site manages approximately 15 ongoing trials annually—predominantly Phases II, III, and IV—spanning a broad spectrum of indications including inflammatory diseases (rheumatoid arthritis, RA; spondyloarthritis, SpA; psoriatic arthritis, PsA), systemic autoimmune diseases (systemic lupus erythematosus, SLE; systemic sclerosis, SSc; giant cell arteritis, GCA; vasculitis, lupus nephritis), and metabolic and bone disorders (gout, osteoarthritis, osteoporosis). Furthermore, in collaboration with the Clinical Pharmacology Department, we extend our research capabilities to Phase I trials. Beyond pharmaceutical trials, our portfolio includes the development of medical devices and a significant number of Investigator-Initiated Trials (IITs). This robust research infrastructure is supported by a specialized team of five principal investigators, 12 sub-investigators, and a dedicated team of study coordinators and nurses, all of whom maintain R3-level GCP certification. In recent years, however, a decline in the number of clinical trials conducted in Hungary, i.e., a small number of protocols initiated at Hungarian sites, as well as a decrease in the absolute number of patients complying to study- and protocol-specific criteria and thus, recruited patients, has been observed. While this trend is influenced by administrative and regulatory complexities following the European Union’s Clinical Trials Regulation (Regulation (EU) No 536/2014), it is also likely linked to the evolving therapeutic landscape. The increasing availability of highly effective approved options—including various biological DMARDs and JAK inhibitors—means that many patients, particularly those with rheumatoid arthritis (RA), achieve clinical remission or low disease activity through standard care. This reduced “therapeutic urgency” can limit the pool of eligible participants who are willing to exchange a stable, effective regimen for an experimental protocol. Additionally, Hungary faces growing competition from emerging research markets in Southeast Asia and South America, which often offer larger treatment-naive patient populations and lower operational costs. Locally, the heavy clinical workload in tertiary centers and a lack of dedicated administrative support for investigators further constrain the capacity to initiate new research projects. This declining trend has prompted national and institutional efforts to strengthen the country’s position in international research networks and to enhance trial feasibility and recruitment. In this context, understanding the perspective and experiences of clinical trial participants becomes increasingly important. Patient-reported satisfaction can provide valuable feedback, not only for investigators and study teams but also for sponsoring organizations, and may influence recruitment efficiency, retention rates, and overall trial performance [2,3].

Previous calls to action have emphasized the importance of systematically evaluating patient satisfaction within clinical trials [2]. Such evaluations may improve openness toward trial participation, support enrollment processes, facilitate achievement of target recruitment numbers, enhance retention, and potentially inform future trial design [2,3]. Reports show that patient satisfaction in clinical practice correlates most highly with interaction with nurses, while patient satisfaction in clinical trials correlates with the study team [2]. Despite these recognized benefits, formal assessment of patient satisfaction in clinical trial populations has remained limited. Existing reports are scarce [4,5,6] and typically consist of literature reviews [7] or pilot studies using simplified Likert-scale instruments [3,8]. One study evaluated 26 trials, where it was found that prior expectation and attitudes toward healthcare and research have an impact on satisfaction [9]. In routine healthcare settings, patient satisfaction is regularly measured in many countries and institutions [10], including the University of Debrecen’s Clinical Center, and overall patient satisfaction with routine care has increased over years [10], yet analogous structured evaluations within clinical trial populations are rarely implemented. Furthermore, there are prior reports and literature reviews on patient satisfaction in general [11], while prior research suggests that determinants of satisfaction may differ between routine care and research settings, so comparative data remains limited. In other therapeutic areas, such as oncology, research has suggested that trial participants often report higher satisfaction due to more frequent contact with specialized staff and a perception of receiving “premium” care [12]. In rheumatology, existing studies have primarily focused on the motivations for enrollment—such as altruism or access to biologics—rather than a direct comparative analysis of the patient experience against standard outpatient care [13]. Consequently, comparative data evaluating patient experiences in clinical trials versus routine specialist care has remained limited, particularly in rheumatology where the long-term nature of the physician–patient relationship may uniquely influence these perceptions.

Given the relative scarcity of structured assessments of trial participant satisfaction and the evolving landscape of clinical research in Hungary, we have sought to investigate this specific patient population. The primary objective of this study has been to assess the opinions, motivations, and experiences of individuals participating in clinical trials in a tertiary rheumatology center. The secondary objective has been to compare experiences of trial participants with their experiences in routine specialist care. The tertiary objective has been to compare perceptions between clinical trial participants and patients receiving standard care only. Finally, we have aimed to evaluate the motivations and perspectives of healthcare professionals involved in clinical research, including principal investigators, sub-investigators, and treating physicians.

## 2. Methods

### 2.1. Study Design and Patient Population

A single-center cross-sectional study was conducted, where a self-administered patient questionnaire was developed in accordance with the study objectives. The questionnaire predominantly used a 6-point Likert scale (Strongly agree, Agree, Neutral, Disagree, Strongly disagree, Not relevant). It was distributed between May 2025 and November 2025 to patients attending specialist outpatient clinics (control population) and to patients currently participating or who had previously participated in clinical trials (study population) at the Department of Rheumatology and Immunology, University of Debrecen, Hungary. Participation was voluntary and anonymous. The study population completed a 21-item questionnaire, while the control population received a 15-item version, excluding items specifically related to clinical trial participation. The questionnaire included demographic questions (sex, age, education level), Likert-scale items, close-ended questions (yes/no), multiple-choice questions (with an “other” option when applicable, e.g., diagnosis), free-text response options, and multi-item questions assessing specific domains. The questionnaires were self-administered. Assistance was permitted in cases of reading or comprehension difficulties; however, helpers were instructed not to influence responses. Participants were informed that their responses were anonymous and would not affect their ongoing medical care or therapy, thereby minimizing social desirability bias.

Additionally, a separate 12-item questionnaire using a Visual Analog Scale (VAS; 0–10) was developed and distributed to principal investigators, sub-investigators, and doctors within the department to access healthcare professionals’ perspectives.

The study was approved by the Hungarian Scientific Research Council Ethical Committee (approval No. DE RKEB/IKEB 7169-2025) and was conducted in accordance with the Declaration of Helsinki and its amendments.

### 2.2. Statistical Analysis

Statistical analysis was performed using IBM SPSS 29.0 (IBM, Armonk, NY, USA). Continuous variables were reported as mean ± standard deviation (SD), minimum, maximum, and mode. Categorical variables were presented as absolute frequencies (*n*) and percentages (%). The distribution of continuous variables was assessed using the Kolmogorov–Smirnov test. Nominal variables were compared between groups using the chi-squared or Fisher’s exact test, as appropriate. Continuous variables were analyzed using paired two-tailed *t*-tests. Although free-text responses were not subjected to formal qualitative analysis, they were reviewed. A *p* value < 0.05 was considered statistically significant, while *p* < 0.001 was considered highly significant.

## 3. Results

During data entry, incomplete questionnaires (*n* = 13) were excluded from the analysis. In total, 129 questionnaires with complete responses were obtained. The proportion of excluded questionnaires was low and therefore unlikely to introduce substantial bias. Although the questionnaire included several questions using a 6-point Likert scale, response distributions demonstrated sparse frequencies in extreme categories. To improve statistical robustness, reduce small-cell bias, and enhance interpretability, the Likert responses were collapsed into a three-category scale: “Strongly agree” or “Agree”; “Neutral”; and “Disagree”, “Strongly disagree”, or “Not relevant”.

### 3.1. Patient Characteristics

**Figure 1:**
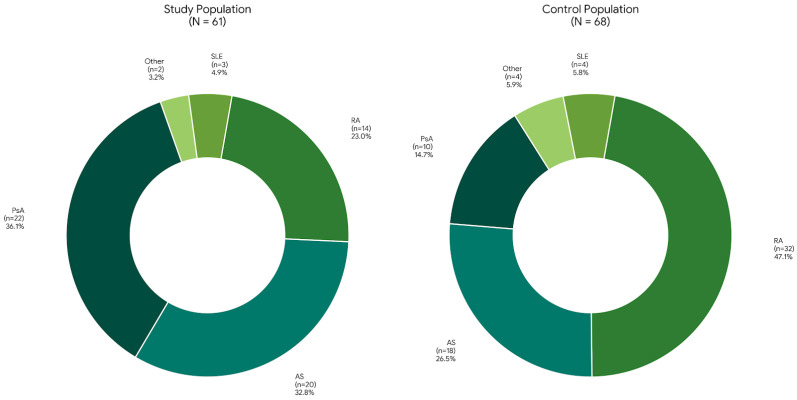
Comparison of diagnostic distribution. Abbreviations: AS, ankylosing spondylitis; PsA, psoriatic arthritis; RA, rheumatoid arthritis; SLE, systemic lupus erythematosus.

A total of 129 patients completed the questionnaires: 61 in the study population and 68 in the control population; additionally, 21 healthcare professionals completed the VAS questionnaire. Patient characteristics are presented in Table 1. The study population included 36 women and 25 men with a mean age of 51.25 ± 13.23 years, while the control population included 41 women and 27 men with a mean age of 53.18 ± 14.37 years. No statistically significant differences were observed between groups regarding sex or age distribution. The study population consisted predominantly of participants enrolled in psoriatic arthritis (PsA) (*n* = 22) or ankylosing spondylitis (AS) clinical trials (*n* = 20), reflecting the current and previous research activity of the department; moreover, other diagnoses included RA (*n* = 14), SLE (*n* = 3), and other (*n* = 2; SSc and vasculitis). Conversely, the control population consisted predominantly of RA patients (*n* = 32) (most patients at the Department), followed by AS (*n* = 18), PsA (*n* = 10), SLE (*n* = 4) and other (*n* = 4; SSc and vasculitis) (Figure 1). Diagnoses included in the control group aimed to reflect those represented in the study population.

No statistically significant differences were observed between groups regarding education level or duration of care at the department. Both groups reported the willingness to participate in clinical trials (study: 90%; control: 79%), both currently and in the future. Prior familiarity with clinical trials differed between groups: 82% of the study population reported no prior knowledge before enrollment, whereas responses in the control group were nearly evenly distributed (51% no prior knowledge, 49% prior knowledge).

### 3.2. Factors Influencing Enrollment in Clinical Trials

Several factors were significantly associated with clinical trial participation, which were significantly different between the study and control population: access to novel therapies; availability of new formulations; more frequent and comprehensive monitoring; access to tests/interventions not available in routine specialist care; health-related questionnaires; recommendation of treating physician; and limited therapeutic options. The strongest motivating factors in both groups were: trust in treating physician (100%, 94%); recommendation of treating physician (100%, 92%); limited therapeutic options (98%, 82%); access to novel therapies (98%, 73%); access to tests/interventions not available in routine specialist care (95%, 69%); and more frequent and comprehensive monitoring (83%, 63%). Interestingly, family members’ advice did not prove to be a motivating factor in either of the groups (54%, 52%) (Table 2). The study population highlighted the recommendation of treating physician (92%) and trust in treating physician (93%), with both showing significant difference when compared to control population (*p* = 0.001 and *p* < 0.001, respectively).

In the control group, the reasons for reluctance included the fear of worsening condition, preference for established treatments, ineffectiveness, and frequent visits, indicating greater reservation within this population. Other studied aspects included ineffectiveness, distrust, attitude of healthcare professionals, family members’ opinions, difficulty in getting to visits, and other (e.g., age was given as an answer).

### 3.3. Patient Satisfaction

#### 3.3.1. Study Population: Clinical Trials vs. Routine Care

The study population reported high overall satisfaction in both settings; however, clinical trials were consistently rated more favorably. The overall satisfaction that was observed with information provided about the disease and therapeutic options; time allocated during the consent process; opportunity for questions; availability of healthcare professionals for questions or complaints; answers given for patient questions; information provided on the upcoming visits, procedures, and medication; and substitution of healthcare professionals if necessary did not significantly differ between the care. Looking in more detail, clinical trials were evaluated to predominantly have the best answers, while answers on routine specialist appointments were distributed mostly between the best and the second best. After investigating this, we found a statistically significant difference in all aspects (*p* < 0.001), except the information provided. Patients of the study population reported overall satisfaction in clinical trials regarding assistance with questionnaire completion and smoothness of visits and examinations, which significantly differed from those during routine specialist appointments (*p* < 0.001) (Table 3).

#### 3.3.2. Routine Care: Study vs. Control Populations

Both groups reported high overall satisfaction during routine care. No significant differences were observed for most general satisfaction measures; however, significant differences were identified in assistance with questionnaire completion (*p* < 0.001) and smoothness of visits and examinations (*p* < 0.05). When analyzed in greater detail, the control population more frequently selected the highest satisfaction category across multiple domains (*p* < 0.001). Information provided on the disease and therapeutic options showed no significant difference (Table 4). Interestingly, we found that the control population perceived the routine care as better as they had no clinical trial experience, compared with the study population, who, after clinical trial experience, had higher expectations regarding routine care.

#### 3.3.3. Satisfaction with Healthcare Professionals

When satisfaction with physician and other healthcare professionals (e.g., study nurse and/or doctors’ assistant) was assessed, the study population reported satisfaction in both the clinical trial, while during routine specialist appointments, no significant difference was observed. When looking into the details, we found that the study population was more satisfied with the team in the clinical trials (*p* < 0.001).

Satisfaction with physicians and healthcare professionals was also assessed during routine specialist appointments and compared between the study population and the control population, where overall satisfaction was observed to have no difference between the groups. When looking into the details, we found that the control population was more satisfied with healthcare professionals during the routine specialist appointments (*p* > 0.001).

Overall satisfaction with clinical trials and routine specialist appointments were also observed, as patients of both groups had the opportunity to ask questions.

#### 3.3.4. Clinical Trials vs. Routine Specialist Appointments

The patient population perceived clinical trials to offer more time for patients per visit; shorter waiting times; calmer environments; more detailed examinations; greater involvement in decision-making; more personalized care; and access to additional interventions. More frequent visits and increased questionnaire burden showed greater variability in responses but were still generally evaluated positively.

#### 3.3.5. Study-Population-Specific Findings

For some study-group-specific questions, e.g., when had they participated in clinical trials, we found that, upon assessment, 18 patients were currently participating, 3 patients had participated within a year, 15 patients within 5 years, 19 patients within 10 years, and 6 patients within 10+ years; however, they had no problem recovering information on their experience. Approximately one-eighth were participating in their first clinical trial. Most patients (93%) had learned about trial opportunities during routine visits. Approximately one-quarter had experienced screening failure due to strict inclusion criteria. Among these, 90% reported disappointment, one-third enrolled in another trial, and nearly all received alternative treatment options promptly.

### 3.4. Healthcare Professionals’ Motivations

In total, 21 healthcare professionals (principal investigator, PI; sub-investigators, SubI; study coordinator, SC) completed the anonymized VAS questionnaire. Positive motivators included availability of new therapeutic options, professional development, financial incentives, while negative motivators included placebo-controlled period possibly lasting up to several months for more serious diseases, time requirements, documentation burden, work outside regular hours, work done cannot be used for scientific advancement, strict inclusion/exclusion criteria, limited eligible patient population, patient compliance, and PI responsibility and associated burden (Table 5).

## 4. Discussion

In this single-center cross-sectional study conducted at a tertiary rheumatology department, we evaluated patient- and physician-reported perspectives on clinical trial participation compared with routine specialist care. Our findings demonstrated that clinical trial participation was associated with high levels of patient satisfaction as reported earlier [8], particularly regarding physician engagement, monitoring intensity, communication quality, and perceived personalization of care. Physician recommendation and trust emerged as the strongest determinants of trial participation, reflecting the central role of the treating rheumatologist in shared decision-making. The importance of trust and communication in influencing patient behaviors has been increasingly recognized in chronic disease management, including RA [14,15].

One of the most consistent findings was the dominant influence of physician recommendation and trust. Nearly all study population patients identified these factors as motivating, consistent with recent work demonstrating that trust in one’s physician influences engagement in care and related decisions [15]. Previous reports showed friendliness as a key factor [8]. Access to innovative therapies and expanded diagnostic or monitoring procedures also strongly motivated participation. This aligned with recent rheumatology research that showed that trial participation decisions were influenced by perceived personal benefit, altruism, and confidence in the care team, with disease-specific variations reported between RA and SLE [15,16]. Specifically, these variations may be attributed to the differing therapeutic landscapes of the two conditions; that is, while RA has a robust selection of approved advanced therapies, SLE has historically been characterized by significant unmet medical needs and fewer targeted treatment options. This often led to a higher therapeutic urgency among SLE patients, who may then perceive clinical trials not just as a research contribution but as a critical pathway to accessing novel treatments unavailable in routine care.

Interestingly, many participants reported limited prior familiarity with clinical trials before enrollment. This mirrored observations that an awareness and understanding of trial opportunities often depended on direct clinician communication, highlighting the importance of structured education integrated into routine care [16,17].

Patients consistently evaluated clinical trial visits more favorably than routine specialist appointments across multiple domains; differences were notable in time allocation, communication, assistance, and perceived thoroughness. This likely reflects structural differences inherent to clinical trial settings, such as protocol-driven monitoring, dedicated research staff, and formalized consent processes—organizational features associated with improved patient-reported experiences across healthcare contexts [18]. Despite higher satisfaction in research settings, overall satisfaction with routine care remained high, suggesting that trial exposure may recalibrate patient expectations rather than reveal deficits in usual care.

Fears of disease worsening, preferences for established treatments, and concerns regarding frequent visits were significant barriers, particularly among the control patients. These concerns echoed themes in broader rheumatology research, where fears of unknown interventions and potential side effects have been cited as deterrents, particularly in SLE, and often interacted with patient risk perceptions [15].

Screening failure due to strict inclusion criteria was associated with disappointment among study participants. The literature has suggested that rigid eligibility criteria may not only limit enrollment but may also negatively affect patient experience and trust, further emphasizing the need for transparent eligibility discussions [19].

From the healthcare professionals’ perspective, access to novel therapeutic options was the strongest motivator for trial involvement. However, documentation burden, time constraints, and the complexity of regulatory processes were deterrents, consistent with recent findings, highlighted the administrative load and resource constraints associated with clinical research in rheumatology [1]. These findings illustrated the dual nature of clinical research involvement, as while it can offer professional enrichment and potential therapeutic advancement, systemic barriers can undermine sustained investigator engagement and patient access.

Recent rheumatology research has underlined the importance of involving patients as research partners in clinical trial design and interpretation. Reviews have shown that patient research partner involvement in randomized controlled trials has remained limited, despite recommendations to increase patient engagement to improve trial relevance and inclusivity. Enhancing patient research partner roles—from trial design to dissemination—may help align research activities with patient needs and expectations, potentially improving recruitment and retention [18].

Our findings suggested that physician–patient relationships were central to successful trial recruitment. Incorporating structured communication about research opportunities into routine care may facilitate informed decision-making and increase participation. Furthermore, elements of trial infrastructure, such as dedicated visit time and systematic monitoring, may inform quality improvement strategies in routine outpatient care, enhancing overall patient experience.

This study integrated both patient and physician perspectives and directly compared clinical trial and routine care experiences in the same institutional context. The inclusion of a control group enhanced interpretability of observed differences; however, several limitations must be noted. First, the single-center design at a university department may limit the immediate generalizability of the findings to the entire nation. While university hospitals often serve as primary hubs for clinical trials, the infrastructure and patient demographics may differ from smaller community hospitals or private practices. Nevertheless, the results remain highly relevant, as the clinical protocols and patient–physician interaction models used here followed national standards. Furthermore, as a tertiary referral center, our patient population had been geographically and socioeconomically diverse, reflecting a broad cross-section of the national population. Other limitations may include a relatively small sample size, a cross-sectional methodology which may preclude longitudinal insights, reliance on self-reported data, and potential selection bias.

## 5. Conclusions

Clinical trial participation in rheumatology has been associated with high patient satisfaction, primarily driven by physician trust, access to innovative therapeutic options, and structured, comprehensive monitoring. Patients perceived study visits to allow more time per consultation and offer a broader scope of care compared with routine specialist appointments, contributing to overall very positive experiences. Although screening failure due to strict eligibility criteria resulted in disappointment, timely identification of alternative therapeutic options by treating physicians mitigated the potential negative impact on patient satisfaction and trust. These findings highlighted the importance of transparent communication [11] and proactive clinical management during the recruitment process. From the healthcare professionals’ perspective, administrative and documentation burdens have remained significant barriers to sustained engagement in clinical research. Addressing these structural challenges will be essential to maintain research activity in academic rheumatology settings. Integrating patient-centered elements of clinical trial infrastructure, such as structured communication, dedicated consultation time, and enhanced monitoring, into routine care may improve overall patient experience. Furthermore, strengthening patient involvement in research design and implementation could enhance recruitment, retention, and relevance in future trials. Finally, the development and implementation of standardized patient satisfaction instruments specific to clinical trials, or even rheumatology clinical trials, would facilitate inter-center comparisons, benchmarking, and systematic quality improvement initiatives, while in order to counter the decline in Hungary, institutions must address the “documentation burden” that currently deters principal investigators.

## Figures and Tables

**Table 1:** Patient characteristics.

Characteristic	Study Population (*n* = 61)	Control Population (*n* = 68)	*p* Value
Female:Male, *n* (%)	36:25 (59:41)	41:27 (60:40)	0.883
Age, mean ± SD, years	51.25 ± 13.23	53.18 ± 14.37	0.407
Diagnosis, *n* (%)			0.084
Rheumatoid arthritis	14 (23)	32 (47)	
Psoriatic arthritis	22 (36)	10 (15)	
Ankylosing spondylitis	20 (33)	18 (26)	
Systemic lupus erythematosus	3 (5)	4 (6)	
Other (SSc, vasculitis)	2 (3)	4 (6)	

**Table 2:** Factors influencing clinical trial enrollment.

Motivation/Influence Factor	Study Population (*n* = 61)	Control Population (*n* = 68)	*p*-Value
Trust in treating physician	100%	94%	<0.001
Recommendation of treating physician	100%	92%	<0.001
Limited therapeutic options	98%	82%	0.008
Access to novel therapies	98%	73%	<0.001
Access to tests/interventions not available in routine specialist care	95%	69%	<0.001
More comprehensive monitoring	83%	63%	0.012
Family members’ advice	54%	52%	0.464
Availability of new formulations	51%	77%	<0.001
Health-related questionnaires	40%	49%	0.029

**Table 3:** Study population: clinical trials vs. routine care.

Domain	Clinical Trial	Routine Care	*p* Value
Information provided about the disease and therapeutic options	98%	92%	NS
Allocated time	97%	36%	<0.001
Opportunity for questions	97%	51%	<0.001
Availability of healthcare professional for questions or complaints	95%	41%	<0.001
Answers given for patient questions	98%	64%	<0.001
Information provided on the upcoming visits, procedures, and medication	98%	66%	<0.001
Assistance with questionnaire completion	93%	36%	<0.001
Smoothness of visits and examinations	98%	34%	<0.001
Substitution of healthcare professional	92%	38%	<0.001

Abbreviations: NS, not significant.

**Table 4:** Routine care satisfaction—study vs. control populations.

Domain	Study Population (*n* = 61)	Control Population (*n* = 68)	*p* Value
Information provided about the disease and therapeutic options	92%	87%	NS
Allocated time	36%	75%	<0.001
Opportunity for questions	51%	81%	<0.001
Availability of healthcare professional for questions or complaints	41%	75%	<0.001
Answers given for patient questions	64%	82%	0.002
Information provided on the upcoming visits, procedures, and medication	66%	84%	0.006
Assistance with questionnaire completion	36%	74%	<0.001
Smoothness of visits and examinations	27%	81%	<0.001
Substitution of healthcare professional	38%	81%	<0.001

Abbreviations: NS, not significant.

**Table 5:** Healthcare professionals’ motivations—VAS scores (0–10).

Factor	Mean ± SD	Mode	Range	Interpretation
Access to novel therapies	8.86 ± 1.26	10	5.3–10	Major motivator
Professional development	7.30 ± 1.95	9.4	4.5–10	Positive
Financial incentives	5.02 ± 2.50	5	0.7–10	Positive
Placebo-controlled period	6.91 ± 3.20	10	0.9–10	Deterrent
Time requirements	5.39 ± 2.86	5	0.8–9.1	Deterrent
Documentation burden	6.69 ± 3.32	8.8	0.9–10	Deterrent
Work outside regular hours	6.08 ± 3.04	5	1.1–10	Deterrent
Work done cannot be used for scientific advancement	3.19 ± 3.19	0.3	0–9.6	Deterrent
Strict inclusion/exclusion criteria	5.03 ± 3.39	2.7	0–9.9	Deterrent
Limited eligible patient population	4.39 ± 2.83	6	0.2–9.7	Deterrent
Patient compliance	3.95 ± 2.29	2.1	0.6–8.8	Deterrent
PI responsibility and associated burden	3.91 ± 2.79	1.5	0–9.4	Deterrent

## Data Availability

Data are available from the authors upon request.
